# Adding Sarcosine to Antipsychotic Treatment in Patients with Stable Schizophrenia Changes the Concentrations of Neuronal and Glial Metabolites in the Left Dorsolateral Prefrontal Cortex

**DOI:** 10.3390/ijms161024475

**Published:** 2015-10-15

**Authors:** Dominik Strzelecki, Michał Podgórski, Olga Kałużyńska, Ludomir Stefańczyk, Magdalena Kotlicka-Antczak, Agnieszka Gmitrowicz, Piotr Grzelak

**Affiliations:** 1 Department of Affective and Psychotic Disorders, Medical University of Łódź, Central Clinical Hospital, Łódź 92-213, Poland; E-Mails: okaluzynska@gmail.com (O.K.); magdalena.kotlicka-antczak@umed.lodz.pl (M.K.-A.); 2 Department of Radiology-Diagnostic Imaging, Medical University of Łódź, Barlicki University Hospital No. 1, Łódź 90-153, Poland; E-Mails: chilam@tlen.pl (M.P.); ludomir.stefanczyk@umed.lodz.pl (L.S.); piotr.grzelak@umed.lodz.pl (P.G.); 3 Department of Adolescent Psychiatry, Medical University of Łódź, Central Clinical Hospital, Łódź 92-213, Poland; E-Mail: agnieszka.gmitrowicz@umed.lodz.pl

**Keywords:** schizophrenia, dorso-lateral prefrontal cortex, glutamate, sarcosine, NMDA receptor, ^1^H-NMR spectroscopy

## Abstract

The glutamatergic system is a key point in pathogenesis of schizophrenia. Sarcosine (*N*-methylglycine) is an exogenous amino acid that acts as a glycine transporter inhibitor. It modulates glutamatergic transmission by increasing glycine concentration around NMDA (*N*-methyl-d-aspartate) receptors. In patients with schizophrenia, the function of the glutamatergic system in the prefrontal cortex is impaired, which may promote negative and cognitive symptoms. Proton nuclear magnetic resonance (^1^H-NMR) spectroscopy is a non-invasive imaging method enabling the evaluation of brain metabolite concentration, which can be applied to assess pharmacologically induced changes. The aim of the study was to evaluate the influence of a six-month course of sarcosine therapy on the concentration of metabolites (NAA, *N*-acetylaspartate; Glx, complex of glutamate, glutamine and γ-aminobutyric acid (GABA); mI, myo-inositol; Cr, creatine; Cho, choline) in the left dorso-lateral prefrontal cortex (DLPFC) in patients with stable schizophrenia. Fifty patients with schizophrenia, treated with constant antipsychotics doses, in stable clinical condition were randomly assigned to administration of sarcosine (25 patients) or placebo (25 patients) for six months. Metabolite concentrations in DLPFC were assessed with 1.5 Tesla ^1^H-NMR spectroscopy. Clinical symptoms were evaluated with the Positive and Negative Syndrome Scale (PANSS). The first spectroscopy revealed no differences in metabolite concentrations between groups. After six months, NAA/Cho, mI/Cr and mI/Cho ratios in the left DLPFC were significantly higher in the sarcosine than the placebo group. In the sarcosine group, NAA/Cr, NAA/Cho, mI/Cr, mI/Cho ratios also significantly increased compared to baseline values. In the placebo group, only the NAA/Cr ratio increased. The addition of sarcosine to antipsychotic therapy for six months increased markers of neurons viability (NAA) and neurogilal activity (mI) with simultaneous improvement of clinical symptoms. Sarcosine, two grams administered daily, seems to be an effective adjuvant in the pharmacotherapy of schizophrenia.

## 1. Introduction

Schizophrenia is one of the most devastating mental diseases, with lifetime prevalence from 0.30% to 0.66% and incidence between 10.2 and 22.0 per 100,000 person-years [1]. It is considered a heterogeneous group of psychoses, caused by a constellation of genetic and environmental factors, with documented heritability [2,3]. A few regions of the central nervous system (CNS) are known to play an important role in pathogenesis of schizophrenia. Mostly reported is the prefrontal cortex (PFC), with its dorso-lateral (DLPFC) and medial (MPFC) regions [4,5]. DLPFC dysfunction is responsible for the negative symptoms of schizophrenia, called “axial symptoms”: autistic behavior, anhedonia and avolition, emotional flattening and social withdrawal [6]. DLPFC also plays a substantial role in cognition, including executive functions that are particularly important in daily life, such as working memory, abstract thinking, task flexibility, planning, and impulse control [7,8]. Cognitive impairment is better predictor of long-term functional outcome in schizophrenia than severity of positive, negative or affective symptoms [9].

From the neurochemical perspective, negative and cognitive symptoms are associated with impairment of the glutamatergic system, especially in the PFC, where ionotropic NMDA receptors are abundant [10,11,12]. Glycine is a necessary co-agonist of the NMDA receptor, and sarcosine (*N*-methylglycine) is an exogenous amino acid that acts as an inhibitor of glycine transporter type 1 (GlyT-1) [13]. Thus, sarcosine should improve the inadequate function of NMDA receptors [12]: a hypothesis confirmed by the observed reduction of schizophrenia symptoms (negative and total symptomatology) associated with the augmentation of antipsychotic therapy with sarcosine [14,15,16,17,18] excluding clozapine [19] or treatment with sarcosine alone [20].

In schizophrenia, there is no consensus on the association between changes in CNS metabolites and exacerbation of symptoms, phase of the disease, treatment strategy or analyzed brain region [21,22,23,24]. Decreased concentrations of *N*-acetylaspartate (NAA), a marker of neuron viability and integrity, are commonly observed [25], and reflect neuronal loss and/or mitochondrial dysfunction [26,27]. However, meta-analyses performed by Steen and Brugger [21,22] found that the NAA concentration in the PFC was similar in patients with a first episode of schizophrenia and in the chronic phase of the disease. It was also not affected by the duration of untreated schizophrenia (DUP) [28] and had already decreased during the pre-psychotic period [29]. Concentrations of NAA, glutamic acid (Glu) and glutamine (Gln) are important in the pathogenesis of schizophrenia, however, many studies have failed to confirm any correlation between metabolite concentration and clinical symptoms [30,31,32,33,34,35,36]. Nevertheless, a few studies have noted an association between negative symptoms and concentration of NAA in the PFC, thalamus and anterior cingulate cortex (ACC) [37,38,39,40].

Although the influence of medications was also evaluated spectroscopically, the findings are ambiguous. In some studies, antipsychotics increased levels of NAA after treatment [34,41], while in others, there were no significant changes [28,42,43,44]. It remains unclear if substances modifying glutamatergic transmission cause changes in concentrations of CNS metabolites detectable in spectroscopy.

The aim of the study is to evaluate the influence of sarcosine therapy on the concentrations of NAA, Glx (complex of glutamate, glutamine and γ-aminobutyric acid GABA), mI (myo-inositol), Cho (choline-containing compounds) and Cr (creatine plus phosphocreatine) in the DLPFC of the left frontal lobe in patients with schizophrenia. Our experiment can support new data on the pharmacokinetics, pharmacodynamics and psychopharmacological value of sarcosine, as well as glutamatergic agents in general. It can also reveal new aspects of the role played by the glutamatergic system in the pathogenesis of schizophrenia.

## 2. Results and Discussion

At baseline, spectroscopy revealed no significant differences in metabolite concentrations between the groups (Table 1).

**Table 1:** Comparison of substances concentrations ratios in study groups.

Compared Ratios	Baseline			After 6 Months			Baseline *vs.* after 6 Months	Baseline *vs.* after 6 Months
	Sarcosine (Mean ± SD)	Placebo (Mean ± SD)	*p*-Level	Sarcosine (Mean ± SD)	Placebo (Mean ± SD)	*p*-Level	Sarcosine *p*-Level	Placebo *p*-Level
NAA/Cr	1.50 (0.65)	1.61 (0.55)	>0.05	1.77 (0.30)	1.69 (0.27)	>0.05	0.0171	0.0468
Cho/Cr	0.75 (0.24)	0.72 (0.38)	>0.05	0.72 (0.15)	0.83 (0.51)	>0.05	>0.05	>0.05
mI/Cr	0.28 (0.13)	0.26 (0.11)	>0.05	0.38 (0.13)	0.28 (0.09)	0.0310	0.0309	>0.05
Glx/Cr	1.10 (0.76)	0.94 (0.30)	>0.05	0.77 (0.29)	0.80 (0.29)	>0.05	>0.05	>0.05
NAA/Cho	2.23 (1.12)	2.08 (0.72)	>0.05	2.61 (0.69)	1.93 (0.58)	0.0061	0.0468	>0.05
mI/Cho	0.43 (0.16)	0.46 (0.64)	>0.05	0.71 (0.25)	0.49 (0.19)	0.0075	0.0064	>0.05
Glx/Cho	0.99 (0.66)	0.81 (0.20)	>0.05	0.99 (0.13)	1.01 (0.34)	>0.05	>0.05	>0.05

NAA, *N*-acetylaspartate; Cr, creatine; Cho, choline; mI, myo-inositol; Glx, glutamate, glutamine and GABA.

In a second spectroscopy NAA/Cho, mI/Cr and mI/Cho ratios were significantly higher in patients receiving sarcosine. Moreover in experimental group after the therapy NAA/Cr, NAA/Cho, mI/Cr, mI/Cho ratios increased significantly, comparing to baseline values.

Only NAA/Cr ratio increased after therapy in the placebo group, although to a lesser extent than in the sarcosine group (4.9% *vs.* 18%).

**Figure 1:**
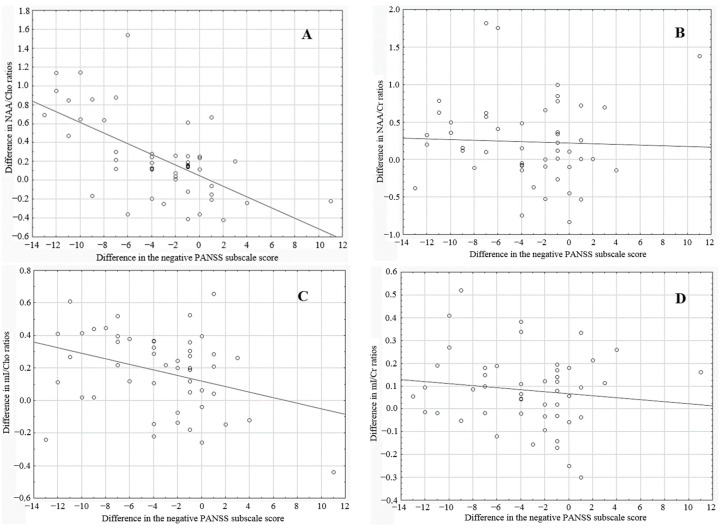
Correlation between the differences in metabolite ratios (**A**) NAA/Cho; (**B**) NAA/Cr; (**C**) mI/Cho; (**D**) mI/Cr and differences in negative PANSS subscale score.

At the beginning of the study, no significant difference was noted between groups with regard to PANSS score (71.4 ± 14 *vs.* 73.3 ± 13 points in total score for sarcosine and placebo groups, respectively; *p* = 0.6736). However, at the end of the experiment, patients treated with sarcosine had significantly lower results (57.7 ± 15 *vs.* 71.5 ± 13 points for sarcosine and placebo group, respectively; *p* = 0.00487). Changes in the negative PANSS subscale followed the trends of the total PANSS subscale. At the beginning of the study there was no significant difference between groups (25.4 ± 5.2 *vs.* 26.1 ± 5 points for sarcosine and placebo groups, respectively; *p* = 0.45085). While the negative PANSS score decreased significantly in both groups (25.4 ± 5.2 *vs.* 18.6 ± 6.1 for the sarcosine group, *p* = 0.0000; and 26.1 ± 5 *vs.* 25.4 ± 4.7 for the placebo group, *p* = 0.03031), this decrease was greater in the sarcosine group (18.6 ± 6.1 *vs.* 25.4 ± 4.7; *p* = 0.00001). The difference in metabolite ratios and negative PANSS subscale scores were calculated between the start-point and end-point of the experiment. Correlations between these differences are presented in Table 2 and in Figure 1.

At the time of writing, this paper was the first attempt to spectroscopically assess the impact of the glutamatergic system modulators, particularly sarcosine, on metabolite concentrations in the DLPFC in patients with schizophrenia. Significant changes in the spectral characteristics co-occurring with alleviation of symptoms, assessed with the PANSS scale, imply that two grams of sarcosine daily sufficiently penetrates the blood-brain barrier to modify the neuronal activity in patients with schizophrenia. Moreover, significant negative correlations between differences in negative PANSS subscale score and spectroscopic parameters (NAA/Cho and mI/Cho ratios) suggest that these ratios might quantitatively correspond with clinical outcomes of therapeutic intervention.

**Table 2:** Correlation between differences in the score of the negative PANSS subscale and metabolite ratios assessed at the beginning and at the end of the experiment.

Differences in Metabolite Ratios Correlated with the PANSS Negative Subscale Score	Spearman’s Correlation Coefficient	*p*-Value
NAA/Cr	−0.130768	>0.05
Cho/Cr	−0.200251	>0.05
mI/Cr	−0.089075	>0.05
Glx/Cr	0.630062	>0.05
NAA/Cho	−0.562891	0.000026
mI/Cho	−0.288039	0.044752
Glx/Cho	−0.200251	>0.05

### 2.1. NAA (N-Acetylaspartate)

*N*-acetylaspartate is one of the most common amino acid in the human brain. It is synthesized in neuronal mitochondria and its production closely correlates with glucose metabolism. Due to the fact that it is not present in glial cells, it reflects neuronal activity well [45].

In our study, both NAA ratios (NAA/Cr and NAA/Cho) in the sarcosine group were significantly higher after six months, indicating an increase of neuronal viability in the DLPFC. In the placebo group, the NAA/Cr ratio was also significantly raised, however, the change was less distinct. Our findings indicate that sarcosine (and probably other GlyT1 inhibitors) might normalize disturbances in brain metabolism and reverse the tendency for NAA levels to decline in schizophrenia. Increased NAA concentrations were also described as an effect of the antipsychotic drugs [34], which may confirm the value of glutamatergic therapy in the management of schizophrenia. Further investigations should assess whether they act synergistically, and if NAA concentration can be used as a marker of clinical outcome.

### 2.2. Glx (Complex of Glutamate, Glutamine and GABA)

An evaluation of Glx level was performed instead of separate glutamine, glutamate and GABA evaluations, as their peaks closely overlap in 1.5 Tesla spectroscopy. Glx is a surrogate of glutamatergic transmission in grey matter as the concentration of glutamate is five times higher than that of glutamine, and 10 times higher than GABA [46].

In schizophrenia, hypofunction of the NMDA receptor may involve GABAergic interneurons, which would result in disturbed glutamatergic transmission [47]. Moreover, there is a decrease of glutamate receptor density on GABAergic interneurons [48]. The summary effect is the inadequate inhibition of glutamatergic neurons, observed in electroencephalography as disturbances of coherent neuronal oscillation at a rate below 0.1 Hz [49] and γ rhythms (25–100 Hz) in PFC [47,50,51,52]. This information noise negatively affects the concentration process and cognitive functions [53,54,55]. Furthermore it can promote hallucinations [56], delusions, and disturbances of the thinking processes and cognitive functions typical of acute psychosis. One of the causes of these pathological conditions is a dysfunction of default mode network (DMN), the “rest system’ of the brain, which should be switched off when working memory networks such as the external attention system (EAS) are activated [53]. In schizophrenia, DMN deactivation is impaired, increasing information noise, intensifying cognitive dysfunction [53,57] and general functioning problems [58].

There is no consensus on the glutamate concentration in the DLPFC of patients with schizophrenia [59]. Kegeles *et al.* showed no significant differences in Glx concentrations between healthy volunteers and groups of medicated and unmedicated patients with schizophrenia [60]. Only three studies have assessed effects of antipsychotics on Glx parameters in the DLPFC before and after treatment. Two studies explored the first episode of schizophrenia: Stanley *et al.* report a decrease in glutamine levels after 14 weeks of antipsychotic therapy [61], and Goto *et al.* note decreased Glx levels in patients after six months of treatment with second-generation antipsychotics [62]. Research conducted in a Polish population showed no changes in Glx levels between baseline assessment and after 40 days of antipsychotic treatment in patients with chronic stage of schizophrenia. However, responders had lower Glx levels at baseline when compared to non-responders [46,63].

On the other hand, the administration of ketamine, an NMDA receptor antagonist whose effect is opposite to sarcosine, resulted in increased glutamatergic transmission in ACC [64,65].

Most studies have failed to find any significant correlation between glutamatergic parameters and PANSS score [46,60,66,67,68]. Kegeles *et al.* report that PANSS positive symptoms subscale scores significantly correlated with levels of GABA and Glx only in MPFC but not in DLPFC [60].

In the present study, a trend was observed towards a decrease of Glx/Cr ratio in both groups. Although it was more expressed in the sarcosine group, the differences were not significant. Further studies using discreet analysis with a stronger magnetic field are required to support more reliable conclusions.

### 2.3. mI (myo-Inositol)

Myoinositol is a precursor in the transmission of phosphatidylinositol, which is a widely accepted glial marker [69]. In neurodegenerative processes, increased mI concentrations co-occur with reduced NAA concentrations.

Significant increases of mI/Cr and mI/Cho ratios in the sarcosine group between two spectroscopies, and in comparison with the placebo group, might indicate unfavourable changes. However, some researchers report greater mI concentrations to be associated with treatment [41,70]. Thus, administration of sarcosine may secondarily activate glial cells, mostly astrocytes, because glycine transporters and other glutamatergic system transporters are abundant in their cell membranes [71].

### 2.4. Limitations of the Study

Due to the limited number of patients and application of 1.5 Tesla magnetic resonance, conclusions should be formulated moderately, as precise separation of glutamate, glutamine and GABA spectra requires a 3 Tesla magnetic field, or higher. Analysis of GABA concentration could be of special interest, because sarcosine indirectly acts on the NMDA receptors located also on GABAergic interneurons. A few studies have found that GABA concentrations varied depending on the analyzed region, including different parts of the frontal cortex [60,72]. On the other hand, prior research has revealed an absence of abnormalities in glutamate or glutamine concentrations in the DLPFC of unmedicated patients with schizophrenia. Thus, the absence of schizophrenia-related glutamate abnormalities in this region may limit the ability to detect a treatment-related change in Glx ratios, which could be detectable in other regions where baseline abnormalities were found, such as the MPFC, striatum, hippocampus or thalamus.

Another important limitation of this work is its application of ratios of metabolites concentrations instead of exact concentrations. Despite changes of Cr and Cho concentrations, depending on duration of schizophrenia, it has previously be demonstrated that treatment with either atypical or typical medication does not alter Cr or Cho levels [73]. Thus, ratios might have a good intra-subject validity [73].

Finally, it should be noted that applied statistical methods did not protect against Type I errors associated with multiple testing. However, although significant differences in particular metabolites could be obtained by chance, the clinical improvement seems to confirm their relevance.

## 3. Experimental Section

Subjects with schizophrenia, aged 18–60 years who were physically, neurologically and endocrinologically healthy and had normal laboratory values (routine blood tests, biochemical tests including thyroid stimulating hormone, lipid profile, liver and kidney parameters) and electrocardiogram were eligible to enter the study. Patients in acute psychosis, on clozapine treatment or declaring suicidal tendencies were excluded from the study. This study is a part of the Polish Sarcosine Study in Schizophrenia (PULSAR); for further details, please see acknowledgments.

Fifty right-handed patients diagnosed with schizophrenia (according to Diagnostic and Statistical Manual of Mental Disorders, 4th Edition, Text Revision (DSM-IV-TR) criteria) with dominant negative symptoms, and who were in a stable clinical condition, were randomly assigned to a sarcosine or placebo group. ^1^H-NMR spectroscopy was performed according to the protocol described below at the beginning of the study and six months later. Sarcosine or placebo were added to the ongoing antipsychotic treatment in a double-blind manner. Patients in the study group were given plastic capsules containing 2 grams of the amino acid, while subjects in the placebo group (similar age, sex, clinical presentation, duration of schizophrenia and treatment, Table 3) received capsules with microcrystalline cellulose. Subjects in both groups were ordered to drink the dissolved contents of one capsule once a day in the morning. All patients were treated with stable doses of antipsychotic and other medication for a minimum of three months before the baseline visit. Doses of antipsychotic and antidepressive drugs were calculated for defined daily dose (DDD) developed by the World Health Organization. Antidepressants were used as a supportive therapy [74] in 14 patients from the sarcosine group and 11 from the placebo group. The differences in the numbers of treated patients and doses in each group were not significant (*p* > 0.05). The severity of schizophrenia symptoms was assessed with the Positive and Negative Syndrome Scale (PANSS) [75].

Subjects were recruited from the outpatient clinic. All patients included in the study have been informed about the aims and methods of the study, and had expressed their written informed consent for participation in this study. The study protocol was approved by the Bioethics Committee of the Medical University of Łódź (permission number and date: RNN/153/08/KE, 15.07.2008). There was no financial involvement from industry.

**Table 3:** Group characteristics.

Features		Group		*p*-Value
		Sarcosine (*n* = 25)	Placebo (*n* = 25)	
Gender	Female	14	12	>0.05
	Male	11	13	
Age (mean)		36.5	40	>0.05
Mean number of hospitalizations		5	4	>0.05
Mean duration of the illness (years)		12.3	13.2	>0.05
Mean timespan of education per patient		14.2	14.4	>0.05
Antipsychotic treatment (DDD)		1.94	1.97	>0.05
Antidepressive treatment (DDD)		0.58	0.6	>0.05
PANSS total (±SD)		71.4 ± 14	73.3 ± 13	>0.05

Abbreviations: *n*, number of patients; DDD, defined daily dose; PANSS, the Positive and Negative Syndrome Scale; SD, standard deviation.

### 3.1. Spectroscopy

Imaging was performed using 1.5 Tesla MR scanner (Siemens Avanto 1.5, Siemens AG, Munich, Germany) equipped with a standard head coil.

NMR acquisition:

(1) FLAIR sequences in axial plane with following parameters: Repetition Time (TR), 9000 ms; Echo Time (TE), 105 ms; inversion time (TI), 2500 ms; flip angle, 150°; voxel size 1.4 mm × 1.3 mm × 3 mm.

(2) T2-weighted sequences were obtained in coronal plane with following parameters: TR = 5000 ms; TE = 100 ms; flip angle, 50°; voxel size 0.6 mm × 0.6 mm × 5.0 mm.

(3) T1-weighted sequences in transverse plane with following parameters: TR = 400 ms; TE = 7.8 ms; flip angle, 90°; voxel size 0.9 mm × 0.9 mm × 0.5 mm.

**Figure 2:**
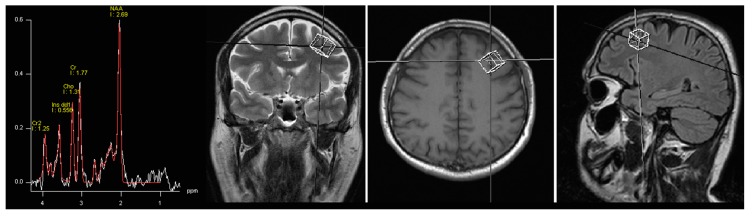
Images showing voxel location in the left DLPFC (dorso-lateral prefrontal cortex) area and an example before (white line) and after (red line) fitting. Peak areas for *N*-acetylaspartate (NAA); creatine (Cr and Cr2); choline (Cho); and myo-inositol (Ins dd1) are labelled.

^1^H-MRS data was acquired by single voxel spectroscopy (SVS) using a point resolved spin echo (PRESS) sequence 128 averages; TR, 3000 ms; TE, 30 ms; voxel size was 15 mm × 15 mm × 15 mm. The region of interest was placed in the left DLPFC by the neuroradiologist (Figure 2). During the second spectroscopy, voxel localization parameters were copied and adjusted to the position of patient. Automated procedures were used to optimize radiofrequency pulse power, field homogeneity, and water suppression, as well as to convert the lines into a Gaussian shape. Spectroscopy data was processed by means of Avanto Syngo MR Software (Siemens AG, Munich, Germany), Level B15. The processing included: k-space Fourier transformation and a spatial 50 Hz Hanning filter; subtraction of the residual water signal; time domain 1 Hz exponential apodization; zero filling to 2048 points; Fourier transformation of the time domain signals; frequency shift correction, phase correction and baseline correction. The fitting error was automatically computed as a deviation between theoretical and measured spectrum determined using the last squares method. Values less than 0.5 were considered satisfactory, however, in the whole group mean fitting error was 0.36 (SD, standard deviation 0.07). The following metabolites were assessed: NAA, Glx, mI, Cho and Cr. No absolute concentrations of metabolites were determined, but their ratios to Cr and Cho.

### 3.2. Statistical Analysis

Continuous variables are expressed as the mean ± standard deviation (SD). The Shapiro-Wilk test was used to determine the normality of the data. As the distribution was skewed in one or both compared groups in all cases, the Mann-Whitney test was employed to compare the ratios of substance concentrations between groups, and the Wilcoxon sign-rank test was used for comparisons within the same group. To evaluate the association between changes in concentrations ratios and differences in PANSS score, the Spearman’s rank correlation test was applied. Statistical analysis was performed using Statistica for Windows (version 12.0, StatSoft, Tulsa, OK, USA). A *p*-value of ≤0.05 was considered significant.

## 4. Conclusions

Our findings demonstrate that addition of sarcosine to antipsychotic treatment can cause increases of NAA and mI in DLPFC. These changes were associated with clinical improvement. It indicates that sarcosine improves neuron viability and integrity, and may activate neuroglial cells in brain regions essential for the pathogenesis of schizophrenia. It highlights the role of glutamatergic transmission in the pathogenesis of schizophrenia and confirms that two grams of sarcosine administered daily may become an effective adjuvant in the management of schizophrenia.
